# The neuroscience of transformers

**DOI:** 10.1007/s11571-026-10566-5

**Published:** 2026-10-08

**Authors:** Peter König, Mario Negrello

**Affiliations:** 1 Institute of Cognitive Science, University of Osnabrück, Osnabrück, 49090 Germany; 2 Department of Neurophysiology and Pathophysiology, University Medical Center Hamburg-Eppendorf, Hamburg, 20246 Germany; 3 Department of Neuroscience, Erasmus MC, Rotterdam, 3015 GE The Netherlands; 4 Department of Bionanoscience, Kavli Institute of Nanoscience Delft, Delft University of Technology, Delft, The Netherlands

## Abstract

Neuroscience has long informed the development of artificial neural networks, but the success of modern architectures invites, in turn, the converse: can modern networks teach us lessons about brain function? Here, we examine the structure of the cortical column and propose that the transformer provides a natural computational analogy for multiple elements of cortical microcircuit organization. Rather than claiming a literal implementation of transformer equations in cortex, we develop a hypothetical mapping between transformer operations and laminar cortical features, using the analogy as an orienting framework for analysis and discussion. This mapping allows us to examine in greater depth how contextual selection, content routing, recurrent integration, and interlaminar transformations may be distributed across cortical circuitry. In doing so, we generate a broad set of predictions and experimentally testable hypotheses concerning laminar specialization, contextual modulation, dendritic integration, oscillatory coordination, and the effective connectivity of cortical columns. This proposal is intended as a structured hypothesis rather than a definitive account of cortical computation. Placing transformer operations and cortical architectonics into a common descriptive framework sharpens questions, reveals new functional correspondences, and opens a productive route for reciprocal exchange between systems neuroscience and modern AI. More broadly, this perspective suggests that comparing brains and architectures at the level of computational organization can yield genuine insight into both.

## Introduction

### The brain and ANNs

The development of artificial neural networks (ANNs) has, at least conceptually, been guided by the biological brain (McCulloch and Pitts 1943). Early neural network models were explicitly framed as abstractions of cortical computation, and contemporary deep neural networks (DNNs)(LeCun et al. 2015) still borrow core ideas such as distributed representations and learning by adjusting synaptic weights (Fukushima 1980; Rosenblatt 1958; Rumelhart et al. 1986). In this sense, the brain has served as an existence proof that large networks of relatively simple units, trained with local updates, can support flexible and powerful cognition. Modern deep learning can be seen as a particularly successful instantiation of this program: by stacking multiple layers of simple, differentiable units and adjusting their connection strengths with gradient-based optimization(LeCun et al. 1998), artificial systems now achieve performance that rivals or even exceeds human performance in certain domains(Hassabis et al. 2017).

At the same time, any close comparison between biological neural networks and standard ANNs reveals substantial differences. The cortex is densely and recurrently connected, with extensive feedback and lateral connections (Douglas and Martin 2004; Harris and Mrsic-Flogel 2013), whereas many of the most successful early deep architectures (e.g., convolutional networks for vision) were predominantly feedforward during inference (Fukushima 1980; Goodfellow et al. 2016). Biological neurons communicate via discrete spikes, operate under tight metabolic and temporal constraints, and exhibit complex dendritic integration (Gerstner et al. 2014); standard ANN units compute continuous, deterministic activation functions, as is typical in mainstream deep learning formulations (Goodfellow et al. 2016). The brain uses a wide variety of neurotransmitters and neuromodulators, implements learning across multiple timescales, and is embedded in a larger architecture of subcortical structures that shape learning, attention, and action selection (Dayan and Abbott 2001). Even at the level of gross anatomy, the common analogy between “layers” in deep networks and cortical laminae, the 6-layered structure of the neocortex, is limited (Mountcastle 1997). Cortical laminae differ in their input–output connectivity, cell types, and biophysical properties(Markram 2006), yet the response properties of neurons within a given cortical column—across different laminae—can be surprisingly similar with respect to classical tuning measures (e.g., orientation selectivity in primary visual cortex) (Hubel and Wiesel 1962). In contrast, artificial networks often implement layers as homogeneous collections of identical units, and functional differentiation across layers is an emergent property of the learned weights rather than reflecting distinct cell classes or canonical microcircuits (Goodfellow et al. 2016). Thus, a multitude of differences between biological and artificial networks might have consequences that are little explored.

These differences are not accidental but the result of deliberate simplifications made for tractability and performance (Marblestone et al. 2016). Deep learning focuses on a small subset of biologically inspired principles—hierarchical feature extraction, distributed coding, and supervised or self-supervised adaptation of synaptic weights—and discards many other aspects of brain organization that are difficult to model or train at scale (LeCun et al. 2015; Kriegeskorte 2015). The success of DNNs in engineering tasks shows that this restricted subset of mechanisms is sufficient to support impressive capabilities, at least in constrained domains, and has in turn provided powerful models for interpreting neural data (Yamins and DiCarlo 2016; Richards et al. 2019).

This became particularly evident in the context of visual object recognition. Deep convolutional networks trained on large-scale datasets such as ImageNet reached and then surpassed human-level performance in object classification (Krizhevsky et al. 2012; Russakovsky et al. 2015). The critical ingredients for this breakthrough were not more biologically realistic dynamics but rather the scaling of network size, the availability of massive labeled datasets, and efficient training procedures on modern hardware (Krizhevsky et al. 2012). Increasing depth, width, and dataset size, together with architectural biases such as convolution and pooling, was sufficient to achieve state-of-the-art results (Simonyan and Zisserman 2015; He et al. 2016; Huang et al. 2017). In this regime, the correspondence between biological and artificial networks was largely metaphorical (e.g., “layered processing similar to the visual hierarchy”) rather than a detailed mapping of neural circuitry, even though such models can be quantitatively compared to neural data in higher visual cortex (Yamins et al. 2014; Khaligh-Razavi and Kriegeskorte 2014).

However, the fact that top-performing models omit many characteristics of real brains does not imply that these biological features are irrelevant to intelligence or to understanding neural computation. Recurrent connectivity, for example, is ubiquitous in the brain and underlies temporal integration, working memory, and context-sensitive processing (Elman 1990; Hochreiter and Schmidhuber 1997); yet recurrence was long underutilized or heavily constrained in many high-performing vision models, despite evidence that recurrent convolutional networks can better capture aspects of biological object recognition (Spoerer et al. 2017). Similarly, spiking dynamics (Maass 1997; Gerstner and Kistler 2002), diverse neurotransmitter systems and neuromodulation (Marder 2012), and interactions with subcortical structures involved in learning and action selection (e.g., reward prediction signals) (Schultz et al. 1997) may be crucial for robustness, energy efficiency, online learning, or flexible behavior in natural environments—properties only partially captured by current ANNs. These aspects have been explored in specialized architectures (e.g., recurrent networks, spiking neural networks, or neuromodulation-inspired models), including work on deep learning with spiking neurons (Pfeiffer and Pfeil 2018), but they are not yet standard ingredients of the most widely used, top-performing systems.

Thus, in the era defined by ImageNet-scale benchmarks, the relationship between biological neural networks and mainstream artificial neural networks remained relatively loose (Kriegeskorte 2015; Hassabis et al. 2017). The brain inspired the initial conceptual framework and some coarse architectural motifs, but the details of cortical anatomy and physiology played little direct role in designing deep learning models that defined the state of the art (Kriegeskorte 2015). At the same time, these models proved useful as quantitative hypotheses about sensory representations (Yamins and DiCarlo 2016) and helped motivate broader frameworks for how deep learning can interface with neuroscience (Richards et al. 2019; Hassabis et al. 2017). This tension—between biological inspiration and engineering abstraction—provides an important backdrop for considering whether newer architectures, such as transformers (see next section), might bring artificial systems into closer alignment with our understanding of the brain, while also addressing limits of current deep learning paradigms (Marcus 2018) and keeping in view the broader methodological role of models in network neuroscience (Bassett et al. 2018).

### The revolution brought by the transformer architecture

The advent of the transformer architecture has fundamentally reshaped the landscape of artificial neural networks (Vaswani et al. 2017). Introduced only a few years ago, transformers have rapidly become the dominant paradigm for sequence processing (Devlin et al. 2019; Brown et al. 2020) and, increasingly, for vision and multimodal integration (Dosovitskiy et al. 2021). In many domains, they have delivered dramatic performance gains, enabling models that generate fluent text, translate between languages, solve complex reasoning problems, and integrate information across modalities at scales and levels of robustness previously unattainable (Brown et al. 2020). These advances have sparked intense discussion not only within machine learning and computational linguistics but also in the wider public sphere, where transformer-based models are increasingly perceived as a step change in artificial intelligence capabilities, including through the framing of such systems as “foundation models” (Bommasani et al. 2021). In contrast to earlier deep learning breakthroughs around convolutional networks and ImageNet, which were largely confined to expert communities, transformer models have quickly become a focal point of societal debate, including prominent critiques of scale and downstream harms (Bender et al. 2021).

Crucially, this leap in performance stems from a significant architectural change rather than merely scaling up existing designs. The transformer replaces the recurrent and convolutional backbones that dominated earlier sequence models (Bahdanau et al. 2015) with a self-attention mechanism (Vaswani et al. 2017). A defining feature of self-attention is a multiplicative interaction among different parts of the input: each input element (e.g., a token in a sentence) generates query, key, and value representations, and the relevance of one element to another is computed via dot products between queries and keys (Vaswani et al. 2017). These multiplicative similarity scores are then used to weight the contributions of the corresponding values. In other words, the model explicitly computes how much “attention” each element should pay to every other element and then integrates information by applying these learned, context-dependent weights (Vaswani et al. 2017). Stacked across multiple layers and heads, this yields a flexible mechanism for contextualizing each representation in light of the entire input sequence, while also motivating work on more efficient attention variants (Choromanski et al. 2021) and enabling straightforward extensions of the same core mechanism beyond language, including vision (Dosovitskiy et al. 2021).

From the perspective of neural computation, this is a striking development. Multiplicative interactions—whether in the form of gain modulation, gating, or context-dependent weighting—have a long but intermittent history in theoretical and systems neuroscience (Salinas and Thier 2000). Models of attention (Treue and Trujillo 1999; Reynolds and Heeger 2009), coordinate transformations (Pouget and Sejnowski 1997), and mixed selectivity (Rigotti et al. 2013) have invoked multiplicative or bilinear terms to explain how neurons combine different sources of information. However, these ideas were typically explored in small-scale, task-specific models and did not crystallize into a widely adopted architectural principle for large, trainable networks. Moreover, the specific structure of transformer-style self-attention—with its separation into queries, keys, and values, its explicit all-to-all interaction pattern, and its central role in high-capacity sequence models—has not been a major focus of prior neuroscientific discussion. In this sense, the transformer architecture introduces a powerful new computational motif into mainstream artificial networks that does not map straightforwardly onto the familiar list of brain–model mismatches (e.g., spiking vs. rate units, recurrence vs. feedforward, cortical vs. subcortical structures) outlined for earlier deep networks. It is not simply a more “brain-like” CNN or RNN; it embodies a qualitatively different way of structuring interactions within the network.

One consequence of this development is that research in computational neuroscience and in state-of-the-art artificial neural networks has, at present, become largely decoupled (Hassabis et al. 2017; Richards et al. 2019). While there is growing interest in using modern deep networks, including transformers, as descriptive models of behavior and brain data (Kriegeskorte 2015; Yamins and DiCarlo 2016), and recent work has reported partial convergence between language models and neural/behavioral measures in NLP settings (Schrimpf et al. 2020; Caucheteux et al. 2022), few neurobiologists treat the transformer architecture itself as a template for understanding cortical or subcortical computation. The dominant theoretical frameworks in systems neuroscience still emphasize recurrent dynamics, attractor states, probabilistic population codes, and canonical microcircuits, rather than attention-like multiplicative routing mechanisms implemented at the scale and form seen in transformers. Conversely, many machine learning researchers who work on transformer-based models do not look to the brain for architectural inspiration in the way earlier generations of deep learning occasionally did (Hassabis et al. 2017). Progress is often driven by empirical scaling, optimization tricks, and engineering constraints, with biological considerations playing at most a peripheral role.

This situation is somewhat paradoxical. At the very moment artificial systems have achieved unprecedented performance across a wide range of tasks, direct comparisons between these systems and the brain have receded from the forefront of both communities. This occurs despite prominent arguments that modern deep learning can be understood as a form of “direct fit” to natural data (Hasson et al. 2020) and ongoing debates about what deep learning does and does not explain (Saxe et al. 2021; Marcus 2018). The transformer architecture has been embraced as an extraordinarily effective tool and has helped motivate the framing of large-scale models as “foundation models” (Bommasani et al. 2021), yet its relationship to known neural mechanisms remains underexplored. Likewise, neuroscience has accumulated a rich body of work on multiplicative interactions, attention, and context-dependent modulation, but has not yet systematically engaged with the specific computational schema that has proven so powerful in artificial models. This growing disconnect raises substantive questions: Are transformers implementing a computational principle that the brain also exploits, but in a different guise? Or do they reflect a fundamentally non-biological solution that is well suited to digital hardware and large datasets, but only loosely related to the mechanisms of biological cognition? In the following, we will argue that closing this conceptual gap—by analyzing transformers through the lens of neuroscience and vice versa—may be essential for understanding these models and for refining our theories of brain function, while keeping in view the broader methodological role (and limits) of models in network neuroscience (Bassett et al. 2018).

## Transformers in the brain

In this section, we move beyond this paradox and outline a concrete, albeit speculative, hypothesis about the relationship between transformers and the brain. Rather than treating transformers as intrinsically “non-biological,” we propose that key aspects of the architecture can be understood as a particular way of organizing computations that the mammalian cortex may already approximate, especially given the long-standing role of multiplicative gain control and normalization in neural computation (Salinas and Thier 2000; Reynolds and Heeger 2009). The central move is to reinterpret the basic unit of comparison while distinguishing representational state from processing substrate. A token is the instantaneous high-dimensional population vector carried by one topographic hypercolumn; the transformer block is the internal vertical laminar microcircuit of that same hypercolumn, principally the $L4\rightarrow L2/3\rightarrow L5$ pathway, which updates the token by routing information over neighboring token states. These complementary mappings are summarized in Table 1. We then ask whether such a block could implement the same kind of context-dependent, gated interactions over shared inputs that define transformer computation (Vaswani et al. 2017).

**Table 1:** Mapping transformer components onto cortical mechanisms (token states within hypercolumn blocks)

Transformer concept	Brain analogue (cortex-centric)
Token	*The instantaneous high-dimensional population vector of a single topographic hypercolumn*; the token is the representational state carried by the module, not the anatomical module itself
Transformer block	*The internal vertical laminar microcircuit of that same hypercolumn* ($L4\rightarrow L2/3\rightarrow L5$), which updates its token via attention-like routing over the token states of neighboring hypercolumns
Input embedding	*Thalamic/subcortical drive* (e.g., LGN*→*V1), providing a shared input embedding to the local circuit
Values (V)	L4*→*L2/3 projection stream, the canonical granule-cell-to-supragranular feedforward pathway.
Queries (Q)	*L1 contextual activity*: long-range feedback projections carrying top-down task goals and predictions that determine which incoming values are retrieved.
Keys (K)	*Tangential streams in laminae 2/3 and 5* that advertise the feature state of neighboring hypercolumns
Attention weights (soft selection)	Ordered operation$A=\textrm{softmax}(QK^\top )$followed by *AV*: *dendritic nonlinearities* generate Q–K compatibility, *inhibitory normalization* produces competitive weights, and the normalized gain then gates Value transmission
Self-attention connectivity pattern	All-to-all (or sparse) token–token interaction; *horizontal (tangential) + feedback connectivity between hypercolumns*, allowing each local block to route information over neighboring population-vector token states
Multi-head attention	Many parallel routing schemes over the same input; *multiple neuronal subpopulations or dynamical modes within each column* sharing the same drive but sampling intracortical context differently (head-like channels whose interactions can span columns)
FFN/MLP sublayer	Local per-token recoding (often expand*→*nonlinearity*→*compress); *local recoding across laminar microcircuits* remapping into a new feature space (“feedforward recoding”)
Residual pathway	Add prior representation with update; *pooling/mixing* with ongoing intracortical + top-down influences
LayerNorm (“Norm”)	Stabilize scale; aid optimization and compositionality; *normalization-like mechanisms* implied via gain control/normalization motifs accompanying attention-like gating (functional, not literal operator)
Stack of blocks	Depth via serial composition (feedforward per pass); *hierarchically stacked cortical modules* with abundant *feedback*; higher modules shape interaction structure in lower modules

More specifically, we will consider whether the internal circuitry of a column can be mapped onto the core components of a transformer block—queries, keys, and values linked by multi-head attention, followed by a local feedforward recoding (Vaswani et al. 2017). On this view, laminar pathways and recurrent loops within a column implement functionally analogous processes to self-attention, while the column’s local input–output transformation plays the role of the feedforward sublayer. We do not claim that this mapping is unique, anatomically literal, or complete; it is offered as an organizing hypothesis and a basis for developing mechanistic models. The departures from a conventional transformer architecture required by this mapping are summarized in Table 2. The aim is to show that once we choose an appropriate level of abstraction, the laminar structure of the cortical column can be viewed as instantiating a transformer-like computational motif, in the same spirit as broader efforts to connect modern deep learning models and neuroscience (Richards et al. 2019) and as proposals emphasizing rich, context-dependent neural representations such as mixed selectivity (Rigotti et al. 2013).

**Table 2:** Relaxed assumptions

Theme	Transformer default	Cortex
Literal equation-level implementation	Exact Q, K, and V matrices plus softmax attention computed as specified	Cortex is hypothesized to implement an *analogous ordered motif* (Q–K compatibility, normalization, then Value weighting), *not* the literal equations token by token
Temporal unfolding	A conventional forward pass is acyclic, with successive transformations across depth	Cortical columns are *strongly recurrent*. One processing cycle may be summarized as an effective transformation; across cycles, representations and effective coupling remain state- and context-dependent
No top-down influence during a pass	Standard transformer block does not receive explicit top-down cortical-style feedback during the same pass	Cortex has ubiquitous *feedback*; top-down + lateral projections extend the module beyond the canonical transformer diagram
Token granularity	Tokens are discrete symbols/patches chosen by a tokenizer/patchifier	A token is the *instantaneous high-dimensional population vector within one topographic hypercolumn*; such vectors vary continuously across cortical maps and interact through long-range connectivity
Input dominance	Input embedding is a major driver of hidden state	Thalamic drive can be a *minor fraction* of synapses (e.g., 10–15% onto recipient neurons), with computation dominated by intracortical context
Explicit heads as separate parameter blocks	Heads are explicit parallel projections with clean vector-space semantics	Heads correspond to *subpopulations* sharing inputs but differing by connectivity and context sampling
Attention as an explicit weight matrix	Attention weights are an explicit, separately computed matrix per head	Gating emerges from *gain modulation + dendritic nonlinearities* (interacting via horizontal and feedback connectivity), not necessarily an explicit “attention matrix”
Single step update	Each layer computes a defined transformation once per step	Cortex supports *iterative refinement* via recurrent/feedback loops; “one box” corresponds to a transformer-like operation embedded in ongoing cycles
Purely local FFN meaning	FFN is a per-token MLP with no explicit anatomical substrate	“FFN” is mapped to *local laminar recoding* and interlaminar transfer; also influenced by top-down integration timing

### A conceptual primer on the transformer motif

To understand how transformer architectures might map onto cortical circuits, it is helpful to contrast them with classical artificial neural networks (ANNs). Traditional feedforward or convolutional networks process data through relatively rigid structural connections, such that an input feature’s influence on subsequent layers is largely fixed after learning. Recurrent neural networks (RNNs) process information sequentially while maintaining a hidden state, which can make long-range dependencies difficult to preserve. By contrast, self-attention allows each element in an input sequence to evaluate the relevance of other elements directly and to route information according to the current input and context.

At its core, the transformer represents inputs as vectors called tokens, such as words in a sentence or patches in an image, and transforms them into updated, context-sensitive tokens. Each token is projected into three functional representations. The Value (*V*) carries the content eligible for transmission. The Query (*Q*) specifies what information the current token seeks, and the Key (*K*) specifies the features by which another token can be matched. These streams arise from the same token but are functionally distinct and not interchangeable. The operation is ordered: Queries and Keys are first compared to form compatibility scores, those scores are normalized to produce an attention matrix $A=\textrm{softmax}(QK^\top )$, and only then are the normalized weights applied to Values to produce *AV*. This non-associative sequence—Q–K matching, competitive normalization, then Value weighting—is the reference computation that the biological mapping must preserve at a functional level.

In our cortical mapping, a token is the instantaneous high-dimensional population vector of one topographic hypercolumn, whereas the transformer block is the vertical laminar microcircuit within that same hypercolumn. The block updates its token by using top-down and lateral context to control how Value-related activity is incorporated. Thus, the anatomical module and the representational token are complementary levels of description rather than competing assignments.

### Elements of the mapping

A starting point is to reconsider the standard mapping between brains and artificial networks. In most comparisons, an entire cortical area is loosely equated with a single “layer” in a deep network (LeCun et al. 2015; Felleman and Van Essen 1991), and the rich laminar structure within that area is collapsed into a single homogeneous set of units, despite extensive evidence for systematic laminar and circuit-level organization in cortex (Mountcastle 1997; Douglas and Martin 2004; Harris and Shepherd 2015). All neurons within a cortical column—across laminae 2/3, 4, 5, and 6—are, in effect, reduced to a single ANN unit or a uniform population within one artificial layer. This is a drastic simplification. Here, we propose that this simplification misses an essential aspect of cortical circuitry and hinders understanding transformer-like computation in the cortex. Instead, what we usually call a “layer” in an ANN should be thought of as corresponding to a module built from several cortical laminae and their recurrent loops, not necessarily to a single anatomical lamina or to a whole area. In this view, the laminar circuit within a cortical column is not an implementation detail that can be ignored but a substrate that realizes a transformer-like operation.

**Fig. 1:**
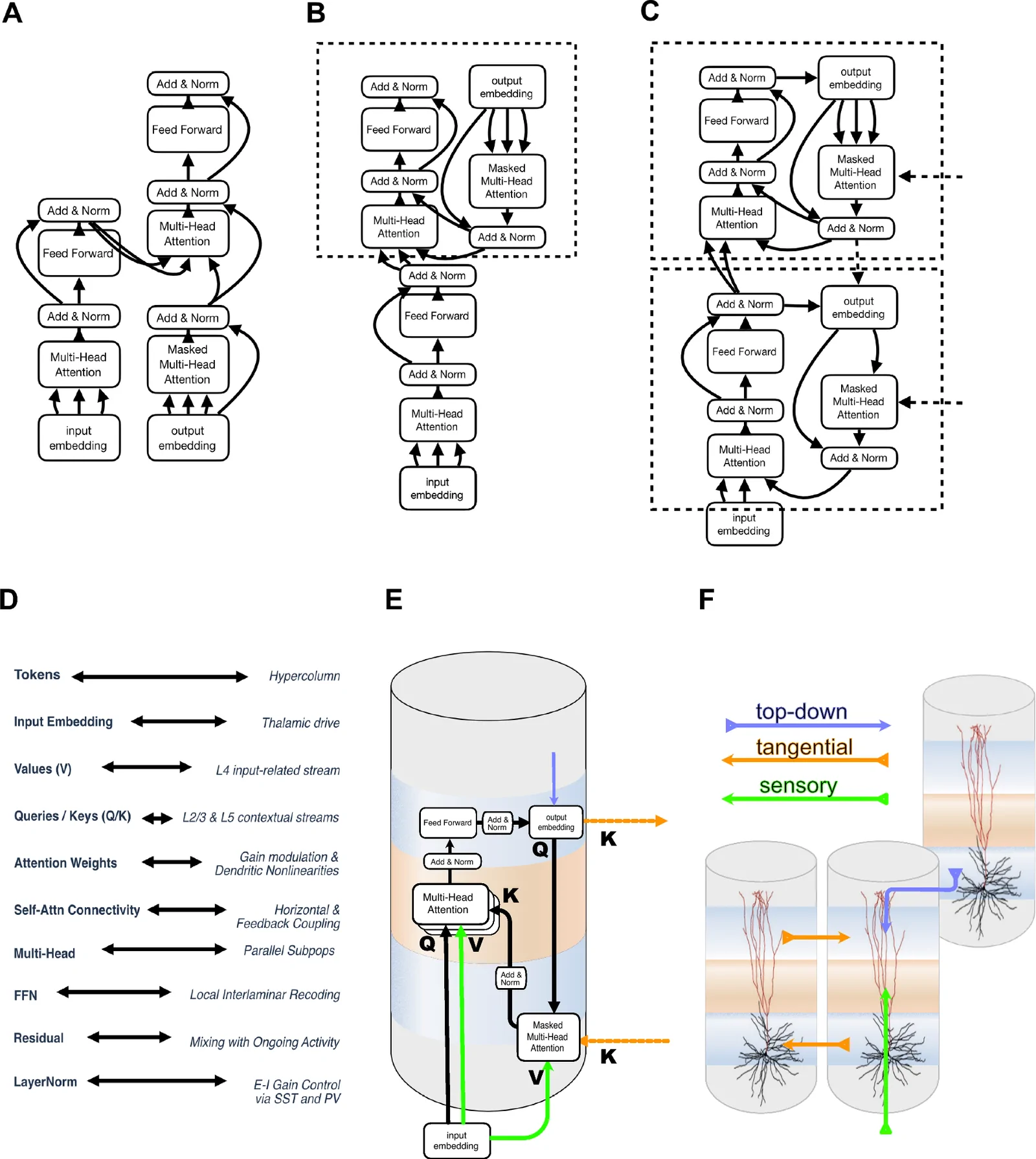
Mapping the transformer encoder/decoder architecture onto the laminar structure of the cortical column. **A** Standard transformer architecture (Vaswani et al. 2017), comprising an encoder stack (Multi-Head Attention *→* Add & Norm *→* Feed Forward *→* Add & Norm) and a decoder stack (Masked Multi-Head Attention *→* Add & Norm *→* cross-attention *→* Add & Norm *→* Feed Forward *→* Add & Norm *→* output embedding). The decoder is conditioned on encoder output via cross-attention. **B** When the decoder stack is inverted vertically, its processing sequence aligns naturally with the laminar depth axis of cortex: deep layers (L5/6) correspond to the decoder-like autoregressive component, whereas superficial layers (L2/3) correspond to the encoder-like component. The dashed boundary marks the operations proposed to occur within a single cortical column treated as a combined encoder/decoder module. **C** The canonical arrangement in (B) extended into a recurrent, stackable single-column module by supplying complementary processing stages. This is a biologically motivated modular generalization, not a purely diagrammatic rearrangement guaranteed to preserve the original input–output function. Repetition of this module across areas would correspond to hierarchical unrolling of the transformer (e.g. V1*→*V2*→*V4*→*IT), with feedforward projections carrying encoder outputs upward and feedback projections carrying decoder/context signals downward. The recurrent arrow denotes influence of the module’s prior output on a subsequent processing cycle. This is a functional analogy to autoregressive conditioning, not a claim that recurrence within one cortical column is mathematically equivalent to traversing an untied transformer stack. **D** Proposed correspondence between transformer components and cortical circuitry. A *token* is the instantaneous high-dimensional population vector carried by one topographic hypercolumn, whereas the *transformer block* is the internal vertical laminar microcircuit ($L4\rightarrow L2/3\rightarrow L5$) within that same hypercolumn. The block updates its local token by routing information over neighboring hypercolumn token states. *Input embedding* corresponds to thalamic drive to L4. *Values* (V) correspond to the canonical L4*→*L2/3 feedforward pathway. *Keys* (K) correspond to tangential L2/3 interactions mediated by horizontal collaterals of supragranular pyramidal neurons. *Queries* (Q) correspond to contextual feedback terminating in L1 on distal apical tufts of L2/3 and L5 pyramidal neurons. The ordered attention computation is $A=\textrm{softmax}(QK^\top )$ followed by *AV*: dendritic coincidence generates Q–K compatibility, inhibitory circuitry approximates competitive normalization, and the resulting gain gates Value-related activity. *Self-attention connectivity* corresponds to horizontal and feedback coupling within and between areas. *Multi-head attention* corresponds to parallel functional subpopulations or dynamical modes within each column whose routing fields extend across neighboring columns. *FFN* corresponds to local interlaminar recoding within the column. *Residual* corresponds to persistent recurrent activity maintaining an ongoing representational state. *LayerNorm* corresponds to E–I gain control mediated by inhibitory interneuron circuitry. **E** Proposed laminar transformer block within a single cortical hypercolumn. Sensory drive ascends from thalamus to L4, forming the input embedding and supplying Values (*V*) to the L2/3 encoder-like stage. Keys (*K*) are conveyed tangentially through L2/3 horizontal projections, broadcasting the hypercolumn’s current population-vector token to neighbouring hypercolumns while receiving their token states. Queries arrive as contextual input to L1 apical dendrites. Query–Key coincidence first generates compatibility signals; local inhibitory circuits then normalize these signals competitively; only then does the resulting gain weight the Value stream before local feedforward recoding. The encoded representation is subsequently passed to L5, where recurrent state conditions decoder-like processing on prior activity; this analogy does not require a literal causal mask. The L5 output constitutes an updated local token state that is broadcast laterally and propagated onward through the cortical hierarchy. **F** Inter-columnar interactions proposed to implement self-attention, multi-head-like computation, and recurrence across the cortical sheet. Top-down signals arriving in L1 provide a shared query broadcast to multiple columns. Tangential L2/3 connections distribute keys laterally according to the structured patchy connectivity of cortex, linking columns with related feature preferences. Thalamic input to L4 provides column-specific values. Distinct functional subpopulations or dynamical modes within each column provide parallel head-like channels, while horizontal and feedback coupling coordinate each channel across the relevant set of columns. Reciprocal tangential interactions provide one possible step of recurrent processing, while top-down feedback can participate in longer loops across the cortical hierarchy. In both cases, recurrent influences undergo the proposed attention-like multiplicative gating rather than simple additive recirculation: Q–K compatibility is normalized before it weights Value-related activity. Recirculated activity can therefore alter both representations and effective gates on the next cycle.

Figure 1 develops this idea step by step. Panel 1 A reproduces, in simplified form, the original encoder–decoder transformer from “Attention is all you need” (Vaswani et al. 2017): on the encoder side, an input embedding is processed by a stack of blocks, each consisting of multi-head attention followed by Add & Norm, then a feedforward sublayer followed again by Add & Norm. On the decoder side, an output embedding passes through masked multi-head attention and Add & Norm, then a second multi-head attention (attending to the encoder output) with Add & Norm, and finally a feedforward sublayer with Add & Norm. In Panel 1B, this architecture is redrawn without changing its computations. The encoder and decoder blocks are rearranged and grouped so that the sequence “multi-head attention *→* Add & Norm *→* feedforward *→* Add & Norm” appears as a recognizable processing unit, and the corresponding decoder sequence “masked multi-head attention *→* Add & Norm *→* multi-head attention *→* Add & Norm *→* feedforward *→* Add & Norm” is laid out to make its internal structure more transparent. This kind of schematic reorganization is also useful for relating the original sequence-to-sequence transformer to later encoder-only variants used in language modeling (Devlin et al. 2019) and to the broader family of transformer architectures now deployed well beyond NLP, including in vision (Khan et al. 2022). At this stage, no new components are introduced; the circuit is only reorganized.

Panel 1 C makes explicit a structural feature that is implicit in the canonical diagram. For our purposes, we introduce the term module to denote a self-contained subcircuit that (i) establishes an input embedding, (ii) applies its own multi-head attention to that embedding, (iii) passes the result through a local feedforward transformation, and (iv) exposes a clearly defined output embedding, with residual and normalization operations wrapped around these stages (Vaswani et al. 2017). A module in this sense is designed to be reusable: it can be instantiated many times in parallel (for example, across a topographic map) or stacked in depth (for hierarchical processing), without requiring additional, bespoke interconnection machinery beyond its standard input–output interfaces.

When mapping the transformer architecture onto neocortical anatomy, an important distinction must be made regarding scale. We propose that a single local cortical module or hypercolumn carries one token as its instantaneous high-dimensional population vector while embodying a complete transformer block in its internal microcircuitry. The token and the block are therefore not mutually exclusive anatomical assignments: the former names the current representational state, whereas the latter names the vertical laminar machinery that updates that state. In its simplest form, the block follows the pathway $L4\rightarrow L2/3\rightarrow L5$, with L5/6 recurrence and feedback further shaping subsequent updates. To achieve context sensitivity, attention-related subpopulations within a given hypercolumn use long-range tangential projections in Laminae 2/3 and 5 to access and weight the token states of neighboring hypercolumns across the cortical sheet. Thus, the computation is locally organized by each hypercolumn’s block but relationally defined over a distributed context window of other hypercolumn tokens.

When we examine the original encoder–decoder architecture of “Attention Is All You Need” through this lens (Vaswani et al. 2017), some functionally paired operations—for example, an attention transformation and its subsequent feedforward recoding—do not fall neatly within a single repeated laminar unit. In that limited schematic sense, the canonical diagram spans approximately one and a half of our proposed modules. Panel 1 C therefore introduces a deliberate modeling extension: it completes the repeated pattern so that each schematic module contains attention, feedforward recoding, residual pathways, and normalization. This completion is not an exact algebraic redrawing of the original architecture; rather, it preserves the ordering and functional roles of the transformer motif while imposing the modular symmetry required for comparison with a repeating cortical microcircuit. The resulting modules can be arranged serially or in parallel, consistent with the general role of residual pathways in stabilizing deep architectures (He et al. 2016) and with interpretations of transformer feedforward sublayers as learned memory-like components (Geva et al. 2021).

Now we take the crucial, and admittedly speculative, step of mapping the elements of one such module onto the laminae of a cortical column (Fig. 1D and E). Here, different cortical laminae are aligned with different parts of the block: external signals targeting lamina 4 are subject to an input embedding and then provide Values toward the supragranular laminae. Pyramidal neurons in laminae 2/3 and 5 integrate top-down Query-like signals terminating in L1/apical compartments with lateral Key-like signals conveyed through tangential projections within canonical cortical circuitry (Douglas and Martin 2004; Harris and Mrsic-Flogel 2013); local dendritic nonlinearities generate compatibility signals, inhibitory normalization makes those signals competitive, and the resulting gain weights Value-related activity (Salinas and Thier 2000; Reynolds and Heeger 2009; Larkum 2013). The dashed arrows in the figure indicate additional top-down and tangential projections that allow the local block in one hypercolumn to operate over token states carried by other hypercolumns. These connections are ubiquitous in the cerebral cortex and naturally extend the canonical transformer motif with feedback and lateral modulation, in line with common canonical-microcircuit perspectives and predictive-coding-inspired accounts of laminar message passing (Bastos et al. 2012). The mapping of individual subcomponents to specific laminae is explicitly speculative: the proposal preserves the ordered computational motif while extending the canonical graph to fit a recurrent laminar circuit.

Our claim is not that the cortex literally implements the transformer equations token by token, but that an analogous ordered computational pattern—context-dependent compatibility, competitive normalization, and Value weighting within a layered, recurrent circuit—may operate in both systems, consistent with the broader view that modern deep networks can serve as useful (if imperfect) computational hypotheses about brain function (Kriegeskorte 2015). By reassigning the correspondence between ANN “layers” and cortical modules and interpreting laminar interactions as attention-like gating, the figure shows how the transformer motif can be compared, after a deliberate schematic extension, with the microcircuit of a cortical hypercolumn. In this mapping, the hypercolumn’s instantaneous population vector is the token, whereas its laminar circuitry is the block that receives an input embedding (for example, thalamic drive), applies context-dependent weighting over neighboring token states, and produces an updated local population vector for subsequent routing.

Across the cortex, many of these modules are arranged in parallel, for instance, over retinotopic or somatotopic maps. In the transformer analogy, the population vectors maintained by these modules collectively form the set of tokens over which self-attention operates: different hypercolumns carry states for different positions or feature combinations, and long-range horizontal and feedback connections implement the “who attends to whom” relationships across this population. Within each module, the vertical laminar microcircuit constitutes the local transformer block, and multiple neuronal subpopulations and dynamical patterns can be viewed as implementing different attention “heads,” all drawing on similar external inputs but sampling and combining intracortical context in distinct ways. Thus, attention in our proposal is inherently multi-scale: each hypercolumn’s block updates its own token through laminar loops and gain modulation, while tangential and feedback connectivity supplies relations to token states carried by other hypercolumns. Framed in this way, the laminated structure of the cortical column becomes a plausible instantiation of a transformer-like computational motif, and this, in turn, motivates a more systematic exploration of transformers as candidate models of cortical computation and of cortical architecture as a source of constraints and inspiration for future transformer-like designs, in line with broader proposals to use deep networks as computational hypotheses for neuroscience (Kriegeskorte 2015) while also engaging with ongoing debates about what exactly contemporary deep learning explains about cognition and biology (Hasson et al. 2020; Saxe et al. 2021) and with frameworks for a productive deep learning–neuroscience interface (Richards et al. 2019).

#### Autoregression via physical recurrence

Mapping the transformer motif onto the neocortical column does not imply treating the cortex as a massive feedforward artificial network. During autoregressive generation, decoder-only and encoder–decoder transformers implement an algorithmic recurrence by appending the output token at time *t* to the context used at time *t+1*, even though each individual block remains feedforward within a pass. Our proposal aligns with this iterative principle but differs in its physical implementation. Artificial models maintain past states in an explicit memory buffer and repeatedly apply static layers; the neocortical column instead contains structural deep-to-superficial feedback loops in which activity from *L*5/6 continuously modulates *L*2/3/4. The biological circuit can therefore maintain and revise a self-referential temporal state through its wiring rather than only through externally stored tokens.

It is important to distinguish hierarchical depth from recurrent time. Transformer depth is an ordered composition of generally untied transformations, in which token representations are updated at successive layers. In our cortical mapping, this corresponds to propagation through anatomically differentiated modules or areas rather than repeated application of one homogeneous cortical layer. Let *m* index a module or hierarchical stage and *t* index a recurrent processing cycle; $X_t^{(m)}$ collects the population states involved in its routing operation. Transformations across the hierarchy need not share parameters and can be written schematically as

$$\begin{aligned} X^{(m+1)}=F_{\theta _m}\!\left( X^{(m)}\right) , \end{aligned}$$

where the parameter set $\theta _m$ may differ between modules. Local recurrence, by contrast, reuses anatomical pathways while acting on an evolving state and changing inputs. To allow for state-dependent nonlinear integration, the schematic Q-, K-, and V-like representations are

$$\begin{aligned} \begin{array}{rcl} Q_t^{(m)} & =& \Phi _{Q,\theta _m}\!\left( X_t^{(m)},r_t^{\textrm{td},(m)}\right) ,\\ K_t^{(m)} & =& \Phi _{K,\theta _m}\!\left( X_t^{(m)},r_t^{\textrm{lat},(m)}\right) ,\\ V_t^{(m)} & =& \Phi _{V,\theta _m}\!\left( X_t^{(m)},u_t^{(m)}\right) , \end{array} \end{aligned}$$

where $r_t^{\textrm{td},(m)}$, $r_t^{\textrm{lat},(m)}$, and $u_t^{(m)}$ denote top-down, tangential, and feedforward inputs, respectively. The functions *Φ* may retain their parameters across cycles while producing different representations and local input–output sensitivities. The ordered attention-like update is

$$\begin{aligned} & A_t^{(m)}=\textrm{softmax}\!\left( Q_t^{(m)}\left( K_t^{(m)}\right) ^\top \right) ,\\ & X_{t+1}^{(m)}=R_{\theta _m}\!\left( X_t^{(m)},A_t^{(m)}V_t^{(m)};C_t^{(m)}\right) , \end{aligned}$$

where $C_t^{(m)}$ denotes lateral and top-down context; attention scaling is omitted for clarity. Shared local parameters do not imply fixed Query, Key, or Value vectors. Even a fixed linear projection, $V_t^{(m)}=X_t^{(m)}W_V^{(m)}$, can produce changing Values as the population state evolves. More generally, effective coupling can vary with sensory drive, recurrent inputs, synaptic efficacy, dendritic state, shunting inhibition, oscillatory phase, and neuromodulation. State-dependent effective coupling does not imply independently learned parameters at every cycle: this recurrent refinement is not mathematically identical to an untied transformer stack. The proposed comparison concerns context-dependent routing motifs while retaining differences in parameterization and dynamics (Vaswani et al. 2017; Markov et al. 2014).

Self-attention is also compatible with recurrent processing in artificial architectures. The Universal Transformer iteratively refines token representations using self-attention and a transition function with parameters shared across recurrent depth (Dehghani et al. 2019). This recurrence operates over successive revisions of representations, rather than over successive token positions. It provides a concrete example of attention combined with parameter reuse, without making tied recurrence equivalent to an untied transformer stack or establishing a biological implementation of either architecture.

For a coarse input–output comparison, one recurrent processing cycle may be summarized as an effective transformation; across cycles, representations and effective coupling remain state- and context-dependent. This abstraction does not require convergence to a fixed point: transient, metastable, or cyclic dynamics may themselves carry computation. The full dynamical implementation remains a hypothesis to be tested rather than an assumed equivalence.

### A detailed walkthrough

In this section, we walk through the circuit diagram in Fig 1D–F step by step and relate each component to known features of cortical microcircuitry (Douglas and Martin 2004; Harris and Shepherd 2015). For clarity, we treat each “box” in the figure as the transformer-like block implemented by the vertical laminar circuitry of a single cortical hypercolumn, including its local recurrent connections (Mountcastle 1997); the instantaneous population activity within that box is its token state. As argued above, the schematic reorganization of the canonical encoder–decoder architecture spans approximately one and a half of these boxes before the repeated pattern is completed in Panel 1C. Here, however, we focus on a single completed box and describe its components using terminology natural for both transformers (queries, keys, values, feedforward stages) and cortical columns (granular, supragranular, and infragranular laminae), drawing where useful on canonical-microcircuit and predictive-coding perspectives on laminar message passing (Bastos et al. 2012).

We begin with the subcortical input. In the primate visual cortex, the projection from the lateral geniculate nucleus (LGN) of the thalamus is considered to be the critical input. These afferents primarily target neurons in lamina 4 and, to a lesser extent, those in lamina 6. Anatomical studies have consistently shown that this thalamic drive constitutes only a small fraction of all synapses onto the recipient cortical neurons, with estimates of 10–15% of the total input each neuron receives (Peters and Payne 1993; Ahmed et al. 1994). From an engineering perspective, this is striking: the very neurons that receive the “external” sensory input are, in terms of synapse count, dominated by recurrent input from within the cortex (Binzegger et al. 2004; Douglas and Martin 2007; Martin 2014). We interpret the processing of thalamo-cortical input in Layer 4 of primary visual cortex as constructing an input embedding (Fig. 1E), with much of the ensuing computation arising from internally generated interactions that evaluate and reweight these afferent signals.

The supragranular laminae implement the next step, a multi-head attention-like operation. Its functional realization may vary across the cortical hierarchy. In early sensory cortices, predictable natural-stimulus statistics—including regularities such as collinearity and contour continuation (Betsch et al. 2004)—may reduce the need to recompute every Query–Key relation de novo. In this limiting, “attention-free” approximation, experience-shaped tangential connectivity supplies a relatively stable prior over Q–K compatibility rather than replacing the ordered attention operation altogether (Ko et al. 2011). Current activity and top-down context can still select among or modulate these predisposed relations, after which inhibitory competition must normalize the expressed compatibility pattern before it gates Values. Gamma-band phase alignment between compatible populations provides one candidate dynamical expression of this structurally biased match (Gray et al. 1989; Singer 1999; Fries 2015). Thus, learned wiring can reduce the cost of dynamic matching while preserving the functional order Q–K compatibility, normalization, and Value weighting.

Crucially, this proposal does not require synchrony to be explicitly optimized as an independent variable. Rather, phase alignment may emerge when populations are linked by sufficiently strong and appropriately structured recurrent, horizontal, or feedback connections and are embedded in local excitation–inhibition dynamics that favor oscillatory responses. Models of cortical circuits containing pyramidal, PV, and SOM populations show that cell-type-specific connectivity can generate and regulate gamma-band activity (Tahvili et al. 2026), allowing sufficiently strong sensory or internally generated events to open transient windows of coordinated communication. Under this interpretation, synchrony is not itself the learned Query–Key match, but the momentary dynamical expression of a match already encoded in circuit structure. Compatible populations are more likely to enter a mutually effective phase relationship when activated together, whereas weakly or incompatibly connected populations do not.

The architectural hallmark of modern transformers—Multi-Head Attention (MHA)—has a possible biological analogue in the fine-scale functional and structural diversity of neurons within a single cortical column. While classical models often view the column as a monolithic module of units with identical tuning, detailed physiological mappings reveal substantial scatter in neuronal response properties (DeAngelis et al. 1999; Ringach et al. 2002). Under our framework, this “functional scatter” could provide a substrate for parallel head-like channels: nearby neurons draw on overlapping feedforward Value-related input—manifesting as similar classical receptive fields (Hubel and Wiesel 1962, 1968)—but diverge in their non-classical receptive fields, which are shaped by horizontal and feedback connections (Das and Gilbert 1999; Fitzpatrick 2000). Distinct neuronal subpopulations within the same column could therefore sample different intracortical contexts and instantiate different Query–Key relations (Gilbert and Wiesel 1983). The prediction is not that anatomically discrete transformer heads will be found, but that relatively low-dimensional compatibility channels will selectively route higher-dimensional Value representations and diverge during complex tasks despite appearing redundant under simple stimuli (Cossell et al. 2015).

After the attention-like stage, activity is transformed and recoded by the proposed analogue of the transformer feedforward sublayer. In an artificial transformer, this stage applies a position-wise nonlinear transformation, often expanding and then compressing the representation. In the cortical column, we associate this recoding with supragranular and interlaminar circuitry that remaps activity into a new feature space (Douglas and Martin 2004; Thomson and Bannister 2003). Top-down input is not restricted to a late, post-computational stage: it contributes to Query-related gating during the update and can continue to refine the resulting representation through recurrent feedback (Markov et al. 2014). A partially processed feedforward estimate may nevertheless propagate before recurrent integration converges, consistent with rapid feedforward transmission followed by slower contextual refinement (Lamme and Roelfsema 2000; Self et al. 2013). Thus, the proposed sequence is not “feedforward first, context only afterward,” but an initial feedforward drive whose expression and subsequent recoding are progressively shaped by context.

As the next step, the activity pattern generated in the output embedding influences the infragranular laminae, which in turn project to subcortical targets and other cortical areas. Transfer can occur through direct supragranular-to-infragranular axonal pathways and through the extended dendritic trees of large Layer 5 pyramidal neurons, which integrate distal inputs in upper laminae (Thomson and Bannister 2003). Much of the tangential connectivity is concentrated in supragranular layers, including prominent intrinsic networks in primate and cat visual cortex (Rockland and Lund 1983; Gilbert and Wiesel 1983; Stettler et al. 2002). We therefore treat residual addition and normalization only as functional analogies: ongoing recurrent activity provides a persisting state onto which updates are mixed, whereas inhibitory gain control stabilizes their scale. Long-range horizontal and feedback projections are more specifically assigned to the Query–Key relations that determine context-sensitive grouping across space and features (Angelucci and Bressloff 2006).

The apical dendrites of Layer 5 pyramidal neurons deserve special mention. Physiological and modeling work has highlighted their role as gating or mode-switching elements that integrate distal contextual inputs and modulate the influence of feedforward drive on spike output (Larkum et al. 1999, 2004; Major et al. 2013). Related theories assign apical dendrites roles in context-dependent processing, prediction, and credit assignment (Körding and König 2000; Urbanczik and Senn 2014; Larkum 2013; Phillips et al. 2018; Phillips and Silverstein 2013). In the present account, apical integration is a leading candidate mechanism for Q–K compatibility and gain control, but the abstract mapping does not depend on one unique dendritic implementation. The essential claim is the ordered functional separation of compatibility computation, normalization, and Value weighting within a recurrent laminar circuit. Supragranular and infragranular populations may access different Query–Key spaces because they receive different combinations of local, horizontal, and feedback inputs, and these relations may be shaped by natural stimulus statistics (Körding and König 2001; Einhäuser et al. 2002; Körding et al. 2004).

### Biophysical micro-mechanisms of the gating operation

To address how these abstract computational operations are physically executed within cortical tissue, we propose two distinct, complementary biophysical mechanisms operating at the network and cellular scales.

At the network scale, horizontal interactions between population-vector tokens need not require an explicit, real-time dot product for every feature pair. As a limiting approximation, an effective Q–K compatibility prior can be embedded in the long-range tangential weights of laminae 2/3. These weights may reflect environmental regularities such as the co-occurrence of visual orientations across space, while dynamic spike patterns carry the activity to be routed. When current activity and contextual state match the learned structural prior, interconnected populations are more likely to phase-lock, opening an effective communication channel between the relevant hypercolumns. Synchrony therefore expresses a compatibility relation encoded partly in connectivity; it neither replaces normalization nor constitutes the Value stream itself.

At the cellular scale, the three operations must remain ordered even if they are distributed across compartments, cell classes, or successive phases of a recurrent cycle. First, top-down Query-related input and lateral Key-related input interact in nonlinear dendritic compartments to generate a Q–K compatibility signal, potentially through NMDA or dendritic calcium events. Second, local inhibitory circuitry converts competing compatibility signals into a normalized gain pattern approximating $A=\textrm{softmax}(QK^\top )$. Third, and only after this normalization, the gain pattern modulates the efficacy of the feedforward Value stream, which may arrive on basal or perisomatic compartments of the same or downstream pyramidal populations. Somatic and axonal output then represents the weighted combination *AV*. This staged account preserves the non-associativity of transformer attention without requiring each mathematical operation to occupy a separate cortical layer.

Finally, the circuit closes. Activity shaped by the local self-attention-like interactions, recoded by feedforward-like transformations, and integrated with top-down context is routed onward via long-range projections to other cortical columns and subcortical structures, within a broader distributed hierarchical organization of cortex (Felleman and Van Essen 1991; Hilgetag and Goulas 1796). At the same time, feedback from those downstream targets returns to the original column, influencing the next processing cycle, consistent with known feedforward and feedback pathways and their distinct functional signatures (Markov et al. 2014; Bastos et al. 2015). In the language of transformers, one processing cycle within a hypercolumn’s laminar block updates its local population-vector token using contextual information from other tokens. Stacking modules across cortical areas provides a sequence of anatomically differentiated transformations, whereas recurrent coupling permits temporally repeated, state-dependent refinement. The resulting network may share the attention-like routing motif of a deep transformer while differing fundamentally in its parameterization and dynamics. Thus, by tracing the flow of activity around this loop, we see how the proposed correspondence between transformer components and cortical laminae gives rise to a coherent, closed processing cycle within and between cortical columns, grounded in cortical connectivity constraints and sensory coding considerations (Harris and Mrsic-Flogel 2013).

The hypothesis is deliberately mechanistic enough to yield concrete, testable predictions, summarized in Tables 3 and 4. First, if multiple attention-like heads are instantiated by different neurons or subpopulations within a column that share a common input embedding but differ in how they sample intracortical context, then their response properties should remain relatively similar under simple, feedforward-dominated stimulus conditions but diverge systematically when rich contextual structure or complex tasks are engaged. In particular, highly controlled receptive-field mapping with simple stimuli should underestimate the diversity of tuning that becomes apparent under naturalistic or task-dependent conditions within the same column.

**Table 3:** Detailed mapping with testable predictions

Transformer concept	In-transformer role	Proposed brain analogue	Testable predictions
Token	Representational state participating in attention	Instantaneous high-dimensional population vector within one topographic hypercolumn	Population-state geometry should vary with local input and context; perturbing one hypercolumn should change context-dependent influence patterns across the sheet.
Transformer block	Updates each token using contextual relations	Internal vertical laminar microcircuit ($L4\rightarrow L2/3\rightarrow L5$) within the same hypercolumn	Layer-specific perturbations should dissociate input-payload processing, contextual routing, and output generation while altering how the local token incorporates neighboring token states.
Input embedding	Shared representational drive	Thalamic/subcortical input	Trial-by-trial variability in thalamic drive should explain less variance than intracortical context in many regimes; stronger context domination during ambiguous/naturalistic stimuli.
Values (V)	Routed content	L4 input-related stream	Manipulating feedforward drive (e.g., L4-targeting) should change content available for routing but not necessarily the routing policy; content effects dissociate from contextual gating.
Queries (Q)	Select what to retrieve	Top-down input to L1 integrated by apical dendrites of L2/3 and L5 populations	Context manipulations should modulate response gain/selectivity in L2/3 and L5 more strongly than L4; causal disruption of feedback or apical integration should impair context-sensitive selection.
Keys (K)	Provide matchable addresses	Horizontal and recurrent contextual streams in L2/3 and L5	Similarity structure between columns (functional connectivity) should be reshaped by lateral and feedback state; increased alignment during grouping/segmentation tasks.
Attention weights	Normalize Q–K matches, then gate Values	Sequential dendritic matching, inhibitory normalization, and Value gain modulation	Q–K compatibility signals should precede competitive inhibitory normalization, which should in turn precede or predict modulation of Value-related transmission.
Self-attn connectivity	Defines interaction graph	Horizontal + feedback coupling	Task-dependent sparsification/rewiring of effective connectivity: same anatomy, different effective coupling; perturbing feedback should alter who influences whom more than raw firing rates.
Multi-head attention	Parallel routing policies	Distinct subpopulations or dynamical modes within columns, coordinated across the cortical sheet	Under rich context, subpopulations should show diverging routing profiles (different target influence patterns); diversity should shrink in impoverished stimuli and expand in naturalistic tasks.
FFN/MLP	Local recoding	Interlaminar transformation/local feature remap	Local nonlinear recoding should be measurable as a change of representational basis across laminae; disrupting specific interlaminar pathways should selectively impair feature recombination without removing contextual gating.
Residual (Add)	Preserve + update	Pooling/mixing with ongoing activity	Residual-like persistence predicts partial invariance of representations across short timescales despite strong contextual modulation; evidence of carry-over even when local recoding is strong.
LayerNorm (Norm)	Stabilize scale	Normalization-like gain control	Context-dependent normalization signatures (divisive-like) should correlate with stability of representational geometry; perturbing inhibitory gain control should destabilize coupling/selection.
Stack of blocks	Deep composition	Hierarchical modules with feedback	Higher-area perturbations should change lower-area effective coupling patterns (routing policy) rather than only adding bias; top-down should change selectivity structure earlier in time than expected from feedforward-only models.

**Table 4:** Relaxed assumptions with falsifiable consequences

Theme	Relaxed assumption	What differs in cortex	Testable consequences/empirical handles
Literal implementation	Do not require explicit softmax/QKV matrices	Emergent gating and dynamics	Look for functional equivalence: context-dependent multiplicative effects and routing signatures, not literal computations.
Temporal unfolding	One-shot feedforward is not required	State-dependent recurrent processing	Time-resolved decoding should distinguish refinement toward stable representations from transient or oscillatory trajectories; later activity should reflect feedback-shaped routing.
Parameters across depth/time	Temporal recurrence need not reproduce an untied stack	Differentiated cortical modules; recurrent cycles reuse pathways with changing effective coupling	Compare untied spatial stages, tied recurrent dynamics, and state-dependent gating under matched resource budgets; test which best predicts time-resolved cortical responses.
Top-down absence	Feedback is central	Bidirectional coupling	Opto/TMS/feedback perturbations should reshape effective connectivity and contextual modulation more than feedforward perturbations.
Token granularity	Tokens are not discrete symbols	Population-vector states within hypercolumns	Token representations should vary smoothly over cortical sheets; context can induce nonlocal coupling edges between the hypercolumns carrying those states.
Input dominance	Input may be weak relative to cortex	Context-dominated regimes	Under ambiguity, reducing thalamic drive may have smaller effects than disrupting intracortical loops on behavior/decoding outcomes.
Head separability	Heads are not explicit modules	Distributed subpopulations/modes	Identify head-like modes via clustering/latent dynamics; diversity should be task- and state-dependent.
Explicit attention matrix	No explicit attention matrix needed	Gating emerges	Measure routing with causal influence metrics (stimulation/Granger/transfer entropy/GLM coupling) rather than expecting explicit weight matrices.
One update per pass	Updates are iterative	Ongoing loops	Recurrent models should outperform feedforward models for time-course prediction; repeated passes (reverberation) should improve fit.
FFN locality	FFN is not a literal MLP	Laminar recoding + timing	Layer-specific perturbations should selectively disrupt feature recombination vs contextual selection, enabling dissociations.

Second, if laminar pathways implement something analogous to queries and keys, then context-dependent gain modulation should show characteristic laminar patterns. For example, manipulations of global or top-down context should preferentially modulate activity and synaptic efficacy in those laminae that carry feedback and horizontal connections (putatively “Q/K-like” pathways), while leaving the initial thalamic drive to the granular lamina (putatively “V-like” pathways) comparatively less affected. Such laminar-specific modulation would support the idea that context acts primarily by reshaping interaction structure rather than by uniformly scaling all inputs.

Third, if multi-head attention corresponds to multiple, partially independent routing schemes over the same inputs, one should observe distinct dynamical signatures associated with different heads—such as separable bands or phase relationships in oscillatory activity, or distinct classes of dendritic events on different branches—each selectively coupling particular sets of columns under appropriate task conditions. Failure to find any of these patterns—no increase in within-column diversity under context, no laminar-specific contextual gain, and no separable dynamical channels for routing—would constitute a serious challenge to the proposed mapping between transformer components and cortical microcircuits.

### What does stacking achieve?

So far, we have focused on the computations implemented by a single module: the vertical laminar circuitry of one cortical hypercolumn acts as a transformer block, while its instantaneous population vector is the token updated by that block. In real cortex, however, such modules do not operate in isolation: they are repeated over topographic maps, connected horizontally, and, crucially, stacked into hierarchies with abundant feedback. It is therefore natural to ask what additional computations become possible when many of these transformer-like modules are arranged in depth and interact over time.

In the artificial transformer, stacking is, by design, purely feedforward within a single forward pass: each block receives the output embedding of the block below, transforms it, and passes the result upward, without any explicit top-down signal from higher to lower layers during that pass. By contrast, cortical hierarchies are bidirectionally connected. Higher areas send feedback projections to lower ones on distinct laminar pathways, and these projections can modulate ongoing processing rather than merely reading out its result. Our proposal can therefore be viewed as a biologically grounded extension of the transformer idea, in which the same kind of module participates in a recurrent hierarchy: over short time windows, activity in higher modules is fed back to lower modules and mixed into their recoding stage, thereby shaping which inputs are considered and how they are combined. This perspective is closely related in spirit to predictive-coding and other recurrent cortical models that emphasize top-down influence, but it casts those ideas in the more explicit language of queries, keys, values.

In the proposed architecture, stacking thus plays a central functional role rather than being a mere repetition of similar units. A useful way to understand this is by comparing with more “classical” recurrent networks and hierarchical cortical models (Markov et al. 2014). In standard recurrent architectures, high-level representations can feed back to earlier processing stages, typically through additive modulation of activity; in biological terms, this corresponds to top-down projections that bias the firing rates or gain of neurons in lower areas according to the current context or prediction (Lamme and Roelfsema 2000). Within the transformer-like scheme we propose for cortex, stacking goes a step further: top-down signals from higher modules do not only modulate the overall activity level of neurons in a lower module, but modulate the interaction structure within that lower module. When the output of an upper module is mixed into the recoding of the lower module’s output embedding, it effectively gates the multi-head attention operating there. In other words, the higher module shapes which queries and keys in the lower module are allowed to interact strongly, thereby altering which inputs are bound together and which relations are emphasized. Stacking thus creates a hierarchy in which high-level context does not simply turn lower-level neurons “up” or “down”, but reconfigures the pattern of connectivity by which lower-level information is combined, consistent with views of early visual cortex as a multiscale “blackboard” for cognitive coordination (Roelfsema and de Lange 2016).

The ordered attention operation is $\textrm{softmax}(QK^\top )V$: Query–Key compatibility is computed and normalized before Values are weighted (Vaswani et al. 2017). Stacking allows this operation to be repeated at progressively more abstract levels while feedback from higher modules reshapes the compatibility structure in lower modules. The synchrony-based and dendritic mechanisms described above provide candidate implementations of this recurrent control: learned connectivity and phase alignment can determine which populations communicate, while compartmental integration and inhibition can translate those relations into normalized gain on Value-related activity (Fries 2015; Larkum 2013; Phillips et al. 2015). Thus, stacking adds not only representational depth but also iterative, top-down reconfiguration of routing. The proposed correspondences between transformer operations, cortical laminar compartments, and candidate oscillatory signatures are summarized in Table 5.

**Table 5:** Correspondence between transformer encoder/decoder operations, cortical laminar compartments, and characteristic oscillatory signatures

Transformer operation	Cortical substrate	Oscillation	Band (Hz)	Key references
Encoder self-attention (L2/3)	Supragranular pyramidal neurons	Gamma	30–80	Bastos et al. (2012, 2015, 2020)
Encoder output/feedforward drive	L2/3*→* Higher areas	Gamma	60–80	Bastos et al. (2015)
Decoder masked self-attention (L5)	Infragranular pyramidal neurons	Beta	14–30	Bastos et al. (2012, 2020)
Decoder feedback/top-down prediction	L5/6*→* Lower areas	Alpha/beta	8–28	Bastos et al. (2015, 2020)
Decoder cross-attention (prediction*)	L5 apical dendrite*←* L2/3 collaterals	Gamma–beta coupling	30–80/14–30	Bastos et al. (2012, 2020)
Thalamocortical input embedding (V)	Thalamus*→*L4	Theta/gamma	4–8/30–80	Bastos et al. (2015)

Cross-attention denotes the decoder querying the encoder representation via L5 apical dendrites receiving L2/3 collateral input

### Learning and plasticity

To map the learning of a transformer block onto cortical columns, we must first distinguish the objectives of the Value, Query, and Key pathways. In artificial networks (Vaswani et al. 2017), $W_V$, $W_Q$, and $W_K$ are all optimized by end-to-end backpropagation, although their functional roles differ. Values provide the content to be routed, whereas Queries and Keys determine the routing weights through dot-product comparison and competitive normalization. A biological implementation may therefore require at least two families of local learning rules: input-driven plasticity that constructs Value representations, and coincidence-dependent plasticity that aligns top-down Query-related activity with laterally supplied Key-related activity. Because the brain lacks a known global backpropagation mechanism (Crick 1989; Lillicrap et al. 2020), these objectives must be coordinated through local signals and circuit structure.

Compartmentalized dendritic trees provide one possible local alternative. Distinct synaptic domains on Layer 5 pyramidal neurons can compare contextual and locally generated activity, while burst- and calcium-dependent plasticity can modify the participating synapses. Such mechanisms do not reproduce backpropagation; rather, they offer local eligibility and teaching signals through which Query–Key compatibility and Value transmission could be adjusted on different timescales.

The principal learnable parameters in this account would therefore be the strength and specificity of horizontal and feedback synapses that couple Query- and Key-related populations. Additional cellular and circuit properties–including synaptic kinetics, short-term plasticity, conduction delays, intrinsic excitability, and the gain and time constants of inhibitory circuits–would constrain the frequency range and temporal window within which coordination can emerge. Our present mapping does not specify how these temporal parameters are tuned, nor whether they are adapted directly or arise developmentally from circuit architecture. A fuller account would require an explicit dynamical model showing how learned connectivity produces selective phase alignment and how that alignment influences the transmission of Value-related activity.

Because the Value pathway functions mathematically as a content provider, its biological optimization can draw on established mechanisms of feedforward feature learning. In sensory cortex, bottom-up drive propagating from the thalamus to lamina 4 and then to supragranular laminae can construct descriptive feature representations. The relevant synapses may be sculpted by Hebbian and spike-timing-dependent plasticity (Markram et al. 1997; Bi and Poo 1998) and refined by local predictive or self-supervised objectives (Rao and Ballard 1999; Bastos et al. 2012). Value learning is therefore not assumed to require the same cross-stream coincidence mechanism proposed for Query–Key alignment, although an explicit model will be needed to establish whether these local rules are sufficient to learn useful content representations and coordinate them with routing.

While the Value pathway can rely on feedforward feature learning, Query–Key alignment demands coincidence detection between distinct contextual streams. Thick-tufted Layer 5 pyramidal neurons provide one candidate substrate because their distal apical and more proximal dendritic domains are electrotonically differentiated (Körding and König 2000; Larkum 2013). In the present mapping, top-down input to the apical tuft contributes Query-related activity, whereas lateral and recurrent synapses on apical, oblique, or proximal compartments contribute Key-related evidence. Feedforward Value-related drive remains a functionally distinct stream, arriving through basal or perisomatic pathways in the same or downstream populations; it is not identified with the Key pathway. When apical depolarization coincides with somatic activity, backpropagating action potentials can trigger dendritic calcium plateaus and high-frequency bursts (Williams and Stuart 2002; Larkum et al. 1999; Major et al. 2013). Burst-dependent plasticity can then strengthen synapses whose activity consistently predicts the contextual event (Körding and König 2000, 2001). This provides a local mechanism for learning Q–K compatibility while preserving the subsequent, separately gated transmission of Values.

While early sensory areas may rely strongly on experience-shaped compatibility priors embedded in connectivity, higher association areas face more variable, context-dependent relations that may require continuing refinement of Query–Key alignment. Across this continuum, cortical circuits can combine relatively stable routing priors with dynamic contextual modulation of the synapses and gain states that determine compatibility. We propose that such adaptation is driven by localized coincidence-dependent plasticity rather than global error backpropagation.

Empirical support for the proposal that an effective query–key ($QK^\top$) interaction structure can be learned through experience comes from the alignment among natural-image statistics, cortical wiring, and population dynamics. In primary visual cortex, the co-occurrence statistics of local orientation features in natural videos closely match both the spatial distribution of boutons in long-range horizontal connections and the degree of oscillatory coherence between orientation-selective neuronal groups (Betsch et al. 2004). This three-way alignment suggests that structural tangential connections and dynamic synchrony patterns in V1 are carved by environmental statistics to internalize recurring feature relationships. Although orientation selectivity in V1 provides only one example, it illustrates how exposure to natural sensory input could establish interaction topologies analogous to a relatively stable prior over Q–K compatibility. Such a prior would constrain, rather than replace, the context-dependent compatibility pattern expressed on a given processing cycle.

Computing Q–K compatibility is necessary but not sufficient: the resulting scores must be normalized before they weight Values. Artificial softmax makes attention weights compete and sum to one. A global, instantaneous cortical softmax is unlikely, but lateral inhibition and divisive normalization can provide a local approximation (Reynolds and Heeger 2009). Strongly activated Q–K detector populations can recruit parvalbumin- and somatostatin-positive interneurons that suppress weaker competitors (Kapfer et al. 2007; Silberberg and Markram 2007). The resulting inhibitory dynamics need not reproduce exponentiation exactly; they need only transform raw compatibility signals into a bounded, competitive gain pattern. That normalized pattern must then modulate Value-related transmission. This ordering distinguishes Q–K detection from the subsequent *AV* operation and yields a direct temporal prediction: compatibility-related dendritic events should precede inhibitory competition, which should precede context-dependent changes in Value efficacy.

Artificial transformers use global backpropagation to jointly shape feature extraction, routing, and parallel attention. The cortex may approximate an analogous motif by segregating these functions across anatomical and biophysical substrates: feedforward plasticity constructs Value representations; compartmental and synaptic plasticity aligns Queries with Keys; inhibitory circuits normalize the resulting compatibility signals; and distinct neuronal subpopulations support parallel head-like routing. This division of labor does not establish that cortex implements transformer learning, but it shows that the proposed architecture does not require a literal biological copy of end-to-end gradient descent.

## Discussion

### Implications

Mapping transformer operations onto cortical laminae may cast new light on biological mechanisms that are underused in mainstream artificial networks (Table 1). In our proposal, laminar circuitry is not a decorative anatomical detail but the substrate that separates content transmission, contextual matching, normalization, and recurrent recoding (Douglas and Martin 2004; Harris and Shepherd 2015). This perspective raises specific questions about the contributions of recurrence, spike timing, oscillations, neuromodulation, and subcortical loops, as well as about the experiments needed to falsify the mapping.

### Empirical avenues

Empirical evaluation requires two complementary steps. First, the modified recurrent model must be analyzed to determine whether its proposed Q, K, V, normalization, and recoding components are functionally distinguishable. Second, neural recordings and perturbations must test whether corresponding laminar populations exhibit the predicted representational and causal dissociations. Interventions that alter specific laminar subpopulations or their effective connectivity should therefore be compared directly with matched perturbations of the model.

A particularly attractive testbed is the Wisconsin Card Sorting Test (WCST), in which participants infer a sorting rule from feedback and adapt when that rule changes without warning (Grant and Berg 1948). Because the visible cards remain unchanged across rule switches, the task dissociates contextual selection from sensory content. Under one operational assignment, Query-like activity represents the current rule (color, shape, or number), Key-like activity represents the candidate match attributes of the cards, and Value-like activity carries the selected object, location, or response information. The model predicts that persistence of the previous Query state will accompany perseverative errors, whereas successful switching will be preceded by realignment between Query- and Key-related representations. Perturbing Query-associated feedback should selectively impair rule updating; perturbing Key-associated lateral circuitry should impair attribute matching despite preserved rule knowledge; and perturbing Value-associated feedforward pathways should impair propagation of the selected response. These assignments must first be validated in the model, and failure to observe the predicted double dissociations in neural data would count against the mapping.

### Recurrent connectivity and temporal scaffolding

Recurrent connectivity is already a major research topic in computational neuroscience and machine learning, with work spanning dynamical systems, attractor networks, working memory, and models of reaction times and decision dynamics (Wang 2002; Wong and Wang 2006; Rabinovich et al. 2008). In many of these studies, recurrence is treated in fairly generic terms: recurrent connections are added, and one asks what behaviors emerge or how they alter fixed points and trajectories in state space. In the framework proposed here, recurrence is embedded inside a transformer-like module with explicit roles for queries, keys, and values mapped onto laminae and cell types. This raises sharper questions: which recurrent loops implement something like key–query interactions, and which loops primarily serve to sustain or stabilize value representations over time? How do different forms of recurrence—within layers, across layers, and between modules—affect the effective depth and capacity of the cortical “transformer stack”? Answering these questions could connect the extensive literature on recurrent cortical dynamics more directly to concrete architectural motifs familiar from artificial networks, and it connects naturally to evidence that recurrence is required to capture the representational dynamics of the human visual system (Kietzmann et al. 2019).

Layer depth, autoregressive generation, and cortical recurrent time are distinct notions: the first orders transformations within a forward pass, the second orders successive generated outputs, and the third describes the continuous state evolution of biological circuitry. Causal masking constrains decoder attention during autoregressive generation, but is not required by encoder self-attention. Our analogy relates these forms of processing without treating them as identical. We propose that oscillations may provide temporal sampling windows without redefining the token itself. Gamma-band activity can organize brief periods of effective feedforward communication, and slower theta or alpha rhythms can group or coordinate successive updates (Lisman and Jensen 2013; Bastos et al. 2015). On this view, each gamma-scale packet samples or updates the population-vector token carried by a hypercolumn. We provisionally associate supragranular processing with encoder-like, predominantly feedforward activity and infragranular processing with decoder-like recurrent and feedback activity; gamma and alpha/beta signatures provide candidate, not definitive, markers of this distinction (Bastos et al. 2012, 2020). The key empirical question is whether Q–K compatibility, inhibitory normalization, and Value modulation unfold in a reproducible temporal order across these laminar rhythms.

### Spiking and dynamical extensions

A decisive next step is to instantiate the proposal in a spiking, recurrent model rather than only in a verbal mapping. Such a model should specify which synapses learn Q–K compatibility, how inhibitory circuits normalize competing matches, when normalized gain reaches Value-carrying populations, and whether distinct subpopulations or phase relationships support separable head-like channels. STDP and other causal plasticity rules provide candidate mechanisms (Bi and Poo 1998; Caporale and Dan 2008), while natural-scene statistics and spontaneous activity provide constraints on the connectivity that should emerge (Berkes et al. 2011; Betsch et al. 2004). The model would be informative only if it predicts spike timing, laminar phase relations, and perturbation effects beyond those produced by a generic recurrent network.

### Functional implications for cytoarchitectonic variations

Crucially, a biological “token” in this framework does not correspond to a static semantic entity, such as a single word in a linguistic dictionary. Instead, a biological token is defined as the instantaneous high-dimensional population vector within a local cortical module or hypercolumn, reflecting features extracted through multiple pathways originating from the organism’s present intra- and exteroceptive situation. The token is therefore the state of the hypercolumn, not an alternative to the transformer block: the block is the internal $L4\rightarrow L2/3\rightarrow L5$ microcircuit that updates this state using relations to neighboring hypercolumn tokens. For example, in the primary visual cortex (*V*1), where receptive fields shift systematically across the tissue, a token represents the joint activity pattern of orientation, contrast, and spatial-frequency features within a local hypercolumn processing a shared region of visual space. This view does not require a rigid, classical layout of discrete feature columns; rather, it treats the combined neural population within a local neighborhood as a high-dimensional feature space that characterizes the local structure of the input. The thalamus does not serve as a lookup dictionary of all possible tokens; rather, it acts as a routing conduit supplying raw sensory data or subcortical motor commands that drive the initial states (*V*) of these topographic modules. Higher-order concepts and sequence generation are therefore not stored in isolated modules, but emerge from distributed, joint activation patterns across entire topographic cortical sheets.

Beyond token-level routing, the framework offers a possible interpretation of the structural diversity of the cerebral cortex itself. Korbinian Brodmann classified cortical areas by regional variations in laminar composition, including the expansion of granular Layer 4 in sensory regions and its reduction in agranular motor cortex. A general computational account of how these laminar profiles relate to different functional demands remains incomplete.

It would be premature to map complex cognitive functions directly onto individual modules, but cytoarchitectonic differences across Brodmann areas may reflect different emphases within the proposed routing motif. The expansion of Layer 4 in granular sensory cortex is consistent with greater investment in feedforward, Value-related input processing. Conversely, the relative reduction of Layer 4 and prominence of deep-layer output circuitry in agranular cortex may increase the importance of recurrent, contextual, and action-related transformations. This comparison does not imply that any area implements only one Q, K, or V function; it predicts quantitative shifts in the balance among content input, contextual routing, and output generation.

Treating cytoarchitectonic maps not only as anatomical markers but also as constraints on different routing regimes therefore provides a testable direction for comparative neuro-AI research.

### Subcortical structures

Subcortical structures also deserve a place in any brain–transformer analogy. The basal ganglia have long been modeled as gating actions and controlling which cortical representations are expressed or updated, both in action-selection models and in cortico–basal-ganglia accounts of working memory (Gurney et al. 2001; O’Reilly and Frank 2006). A transformer-informed analysis can sharpen this analogy by asking whether particular loops primarily gate Value-related outputs, Query-related contextual states, or Key-related candidate representations. These alternatives make different predictions about whether perturbations alter propagated content, the active task rule, or the set of matchable representations. Developing those distinctions may connect the present cortical proposal to established functional anatomy and dopamine-based accounts of cognitive control (Lanciego et al. 2012; Frank 2005).

## Alternative approaches

A small but growing body of work has begun to ask how transformers, and in particular self-attention, might be implemented in more biologically grounded circuits. One influential line focuses on neuron–astrocyte networks: Kozachkov and colleagues show that tripartite synapses can, in principle, implement the core components of self-attention, with astrocytic dynamics providing the normalization and multiplicative gating that parallel the softmax attention operator (Kozachkov et al. 2023). They extend this framework to dense associative memory models in which neuron–astrocyte dynamics converge to attention-like fixed points over stored patterns (Kozachkov et al. 2025, n.d.). A second, largely orthogonal line develops spiking and neuromorphic transformers, such as Spikformer and related architectures, which replace rate units with spiking neurons and implement attention using spike-based Q, K, and V representations, sometimes coupled to local plasticity rules so that effective attention weights emerge from synaptic changes rather than explicit matrix multiplications (Zhou et al. 2023; Mondal and Kumar 2025). These approaches demonstrate that transformer-style computations can be realized in biophysically flavored systems—either at the level of detailed synaptic and glial dynamics or at the level of spike-based algorithms aimed at energy-efficient hardware—but they remain relatively agnostic about the mesoscopic architecture of the cortex. In contrast, our proposal takes the laminar cortical column as the primary unit of analysis and asks whether its canonical microcircuitry can be interpreted as a reusable transformer-like block: queries, keys, and values are assigned to specific laminar pathways and recurrent loops; multi-head attention is mapped onto parallel neuronal subpopulations within a column; and self-attention across population-vector token states is realized by horizontal and feedback connectivity between columns. As a consequence, the frameworks differ in emphasis and in their most direct experimental predictions. Neuron–astrocyte accounts imply that disrupting astrocytic signaling or tripartite synapse dynamics should directly impair attention-like normalization, whereas our account predicts strong task- and context-dependent differentiation of responses across laminae and within columns, even when classical receptive fields appear similar under simple stimuli. Likewise, spiking transformer models make distinctive claims about the role of precise spike timing and specific spike-based plasticity rules in implementing attention, whereas our proposal emphasizes laminar patterns of gain modulation and long-range grouping. Taken together, these complementary but competing pictures suggest a rich experimental agenda: by systematically probing spike timing, glial contributions, and laminar-specific contextual effects under naturalistic tasks, it should be possible not only to test whether the cortex implements an attention-like computational motif but also to discriminate between alternative biological implementations of transformer-style computation.

A closely related theoretical proposal by Granier and Senn similarly argues that cortico–thalamic circuits can implement multi-head self-attention, but locates the organization of attention heads at a different spatial scale (Granier and Senn n.d.). Their construction uses cortical microcolumns to realize the internal computation of one attention head and associates parallel cortical areas with distinct heads whose outputs are integrated through thalamic loops. By contrast, our framework treats the mesoscopic cortical column and its intrinsic laminar microcircuit as the foundational transformer-like block, with head-like operations distributed among local neuronal subpopulations. Both accounts emphasize multiplicative integration involving Layer 5 pyramidal dendrites in the generation of context-dependent attention signals, whereas our approach places additional emphasis on local interlaminar recoding and horizontal collateral networks as mechanisms for attention within and between columnar populations.

The proposed mapping also aligns with the Layer 6b Theory of Attention (LAT) (Zolnik et al. 2026). Whereas our framework emphasizes Layer 5 pyramidal integration as a candidate substrate for Q–K compatibility, LAT positions L6b as a conductor of attention-related cortico–thalamic loops. In a biological-transformer interpretation, L6b would not necessarily supply the feature-specific Query or Key. Instead, its projections to Layer 5 apical tufts and its coupling to higher-order thalamus could regulate the gain, persistence, or effective temperature of local competition. This suggests a hierarchical control scheme in which local laminar blocks perform feature-specific routing while the L6b–thalamic system adjusts state-dependent stability and attentional sharpness over longer timescales.

### Transformers in the hippocampus?

While the framework developed in this manuscript focuses exclusively on the canonical microcircuitry of the neocortical column, it is worth noting that the computational motif of the transformer–namely, dynamic, context-dependent routing over shared inputs–is increasingly recognized as a broader organizing principle across other brain structures. Recent computational modeling has begun to explore these parallels in the hippocampal formation, suggesting that auto-associative networks like CA3 and the relational memory mapping of grid and place cells can be mathematically reconciled with self-attention mechanisms and transformer-like configurations (Whittington et al. 2022; Kim et al. 2023; Rolls 2024). Crucially, however, the biological implementation of this motif appears to follow substantively different anatomical constraints. Whereas the neocortex stacks encoding and decoding operations vertically within the dense, localized laminar microcircuitry of a single column, the archicortex (hippocampus) deploys them across a sequential, loop-like macro-circuitry spanning distinct anatomical regions (Entorhinal Cortex *→* Dentate Gyrus *→* CA3 *→* CA1). Our columnar proposal should therefore be viewed not as a unique biological solution, but as a specific, highly parallelized evolutionary implementation of the transformer motif, leaving the distinct macro-loop architecture of archicortical transformers as a separate, complementary line of inquiry.

## Conclusion

In this essay, we have proposed that the mammalian cortex may implement a transformer-like computational motif through its laminar and recurrent organization. This is not intended as a sweeping general theory of brain function in the vein of predictive-coding, information-integration, neural-energy, or large-scale self-organizing frameworks (Rao and Ballard 1999; Tononi 2004; Wang and Zhang 2023; Carpenter and Grossberg 1987); rather, it concerns a possible set of circuit-level building blocks. Many assignments in the present mapping are likely incomplete or oversimplified, and the correspondence between cortical laminae and transformer components will vary across areas and tasks. The central hypothesis is narrower: cortical microcircuits may implement ordered, context-dependent routing in which Query–Key compatibility is computed and normalized before it modulates Value-related activity. Framed this way, the proposal yields concrete computational and experimental tests and may deepen exchange between artificial-network research and systems neuroscience.

## Data Availability

No datasets were generated or analysed during the current study.
